# Early mobility after fragility hip fracture: a mixed methods embedded case study

**DOI:** 10.1186/s12877-021-02083-3

**Published:** 2021-03-15

**Authors:** Lynn Haslam-Larmer, Catherine Donnelly, Mohammad Auais, Kevin Woo, Vincent DePaul

**Affiliations:** Queen’s University, Faculty of Health Sciences, School of Rehabilitation Therapy, Louise D. Acton Building, 31 George Street, Kingston, ON K7L 3N6 Canada

**Keywords:** Embedded case study, Fragility hip fracture

## Abstract

**Background:**

Following a hip fracture up to 60% of patients are unable to regain their pre-fracture level of mobility. For hospitalized older adults, the deconditioning effect of bedrest and functional decline has been identified as the most preventable cause of ambulation loss. Recent studies demonstrate that this older adult population spends greater than 80% of their time in bed during hospitalization, despite being ambulatory before their fracture. We do not fully understand why there continues to be such high rates of sedentary times, given that evidence demonstrates functional decline is preventable and early mobility recommendations have been available for over a decade.

**Methods:**

A descriptive mixed method embedded case study was selected to understand the phenomenon of early mobility after fragility hip fracture surgery. In this study, the main case was one post-operative unit with a history of recommendation implementation, and the embedded units were patients recovering from hip fracture repair. Data from multiple sources provided an understanding of mobility activity initiation and patient participation.

**Results:**

Activity monitor data from eighteen participants demonstrated a mean sedentary time of 23.18 h. Median upright time was 24 min, and median number of steps taken was 30. Qualitative interviews from healthcare providers and patients identified two main categories of themes; themes external to the person and themes unique to the person. We identified four factors that can influence mobility; a patient’s pre-fracture functional status, cognitive status, medical unpredictability, and preconceived notions held by healthcare providers and patients.

**Conclusions:**

There are multi-level factors that require consideration with implementation of best practice interventions, namely, systemic, healthcare provider related, and patient related. An increased risk of poor outcomes occurs with compounding multiple factors, such as a patient with low pre-fracture functional mobility, cognitive impairment, and a mismatch of expectations. The study reports several variables to be important considerations for facilitating early mobility. Communicating mobility expectations and addressing physical and psychological readiness are essential. Our findings can be used to develop meaningful healthcare provider and patient-centred interventions to address the risks of poor outcomes.

## Background

Following a hip fracture, up to 60% of patients are unable to regain their pre-fracture level of mobility [1, 2]. For hospitalized older adults the deconditioning effect of bed rest and functional decline has been identified as the most predictable and preventable cause of loss of independent ambulation [3]. National and international hip fracture guidelines [4–6] recommend several interventions geared towards preventing this hospital-related functional decline, one of which is early mobility after surgery. It has been shown that early mobility can decrease the overall length of hospital stay and aid in re-establishing a patients’ functional status and return to their pre-fracture environment [4]. Recent studies have shown that this older adult population spends greater than 80% of their time in bed during hospitalization, despite being ambulatory before the fracture [7–9]. We do not fully understand why there continues to be such high rates of sedentary times, given that evidence demonstrates functional decline is preventable and early mobility recommendations have been available for over a decade.

In 2011, an interprofessional team of health care providers at a large urban tertiary centre in Toronto, Ontario implemented the Bone & Joint National Model for Hip Fracture Care & Toolkit (2011) on one post-operative unit. The Hip Fracture Care & Toolkit was designed to standardize and disseminate hip fracture best practices across the continuum of care in all Canadian provinces [6]. A recent chart review of the older adult hip fracture population on this unit [10] identified that early mobility activities are initiated in the first five days after surgery to varying degrees. Many patients did not participate in early mobility activities, with non-participation rates higher in those with a low pre-fracture functional mobility level and those with cognitive impairment. The reasoning behind the lack of patient participation was not clear; health care provider behaviours appeared to be in line with recommendation utilization. The chart review failed to provide insight into any hospital-based factors that may influence health care provider or patient actions.

Historically, the implementation of best practice recommendations has focused on changing health care provider behaviours, yet these behaviours (i.e., early physiotherapy involvement) represent only one piece of the complex interchange between the system and patient. Exploring other factors that may influence the facilitation of activities and patient participation is required. There is a paucity of data that explores other factors associated with an older adult’s mobility hesitancy after hip fracture surgery. To develop effective interventions geared towards improving mobility while in hospital, there is a need to understand locally relevant factors. The purpose of this study is to a) describe early mobility activities on one post-operative unit with a history of recommendation implementation and b) identify factors influencing participation in early mobility activities after hip fracture surgery.

## Methods

A descriptive embedded case study design using mixed methods was selected to understand the phenomenon of early mobility after fragility hip fracture surgery. A case study brings together multiple qualitative and quantitative data sources to provide an in-depth understanding of complex phenomena [11]. A convergent mixed-method approach was utilized to interpret this data - collecting quantitative and qualitative data concurrently, weighing these methods equally, and subsequently interpreting the results together [12].

An embedded case study design enables examining multiple units of context within a single main case [13]. In this study, the main case is the post-operative unit, and the embedded units are the patients (and their families) recovering from hip fracture repair. The patients are ‘embedded’ within the case study as recipients of recommendation utilization by health care providers. Obtaining data from multiple sources and key stakeholders provides us with the most comprehensive understanding of mobility activity initiation and patient participation on the selected post-operative unit. The following sections outline the embedded case study research process, with descriptions of elements within both the main case and embedded units.

### Defining, binding, and selecting the cases

#### Main case context

The study took place in a large tertiary care centre located in Toronto, Ontario. The hospital admits approximately 200 older adults with hip fracture per year [14]. As per Health Quality Ontario (HQO) recommendations [15], surgical repair of a fragility hip fracture occurs within 48 h of patient admission [10]. The main case was purposefully selected as the unit where most patients after hip fracture surgery are admitted, as this unit had previously implemented hip fracture recommendations in 2011. Participants within the main case included only the healthcare providers (HCPs) who are directly involved in mobilizing patients and familiar with the study population: physiotherapists, occupational therapists, therapy assistants, and registered nurses. All five therapists on the unit were approached to participate. We invited nursing staff to participate by attending daily nursing rounds and placement of study posters. Within the institution, the physicians are responsible for patient management, for example, ensuring referrals are in place for physiotherapy, and ordering a weight-bearing status that allows for mobility. Physicians are not directly involved in mobilizing patients at the bedside. Data collected included the order sets, but physicians were not approached to participate in the interviews.

### Embedded units – patient cases

The embedded patient cases were bound by three inclusion criteria (time, activity, and place): adults 65 years of age or older, admitted for surgical repair of a fragility hip fracture with post-operative admission to the unit of study, and able to independently ambulate before experiencing the fracture. Older adult patients were excluded if they were unable to walk independently before their fracture, had a traumatic hip fracture or suffered from a concurrent illness that could affect mobility (e.g. stroke), or presented to hospital over 48 h post-fracture.

The Mini-Mental State Examination (MMSE) [16] was utilized at the time of consent to assess cognitive status,. With a total score of 30 points, scores ≥25 points is considered normal, 20 to 24 represent mild cognitive impairment, 11–19 represent moderate impairment, and ≤ 10 severe cognitive impairment [16]. The New Mobility Score (NMS) was used to assess pre-fracture mobility function [17]. For this study, we dichotomized the NMS groups – low (NMS 2 to 5) and high (NMS ≥ 6) [18]. Those with a NMS score less than 2 were excluded (unable to ambulate indoors independently).

Patients admitted to the hospital with a hip fracture were screened in the emergency department by a research team. If the patient had a physician documented history of delirium or dementia on their chart, the substitute decision-maker (family) was approached for consent. For all participants, family members were advised at time of consent that they could also participate in the interview. The consent included a statement that allowed for ongoing participation should delirium occur after surgery.

### Data collection sources

**Fig. 1:**
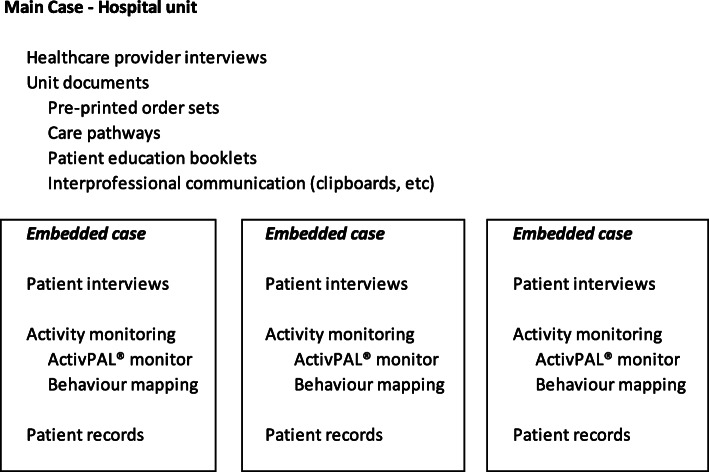
Data Collection Sources

For this study, transitioning from sedentary behaviour (e.g. bed rest) to active physical activity after surgery was the phenomena of interest. We explored this phenomenon using multiple data sources as outlined in Fig. 1.

#### Main case

The main case unit has 38 beds, with 20 patient rooms: 9 private, 4 semi-private, and 7 ward rooms. Therapists confirmed unit room measurements as distances were commonly referred to in their narrative documentation: five metres is the approximate distance to the bathroom, with ten metres reflective of a trip to the bathroom then returning to the bed. If able, patients ambulate in hallways with staff, family or alone, essentially doing ‘laps’ around the unit.

To understand the unit-based influences on mobility, we obtained unit documents (policies, procedures) and interviewed healthcare providers. The healthcare provider interview questions were developed using the Theoretical Domains Framework (TDF) [19]. The TDF was designed to identify areas of gaps in knowledge or identify domains of barriers and enablers of clinician behaviours, such as beliefs and consequences of a clinician’s actions [20]. As an oft-cited validated framework within healthcare implementation research, the TDF comprises 14 domains and 84 constructs identified as influencers on behaviours [19, 21]. As a theoretical framework, it is designed to guide the cognitive, social, and environmental assessment of influences on clinician behaviours; not to propose testable relationships [19]. The number of domains covered and the number of questions within each domain are dependent on the healthcare provider clinicial behaviours being assessed and the existing evidence about these behaviours. This study’s interview guide was developed to assess a healthcare providers’ awareness of early mobility recommendations and further explore their perception of their ability to adhere to recommendations. For example, within the Environmental resource and context domain, one question was “are there any factors on the unit which impact your ability to mobilize patients with hip fracture after surgery? What about those with cognitive impairment – are there any factors on the unit which may act as a barrier or facilitator to mobilization?”

#### Embedded units – patient cases

We obtained participant mobility activity data from a number of sources (Fig. 1). Firstly, participants wore the ActivPAL® activity monitor. The ActivPAL® monitor has been found to be a valid and reliable measure of walking activity in community-dwelling older adults [8]. Using a triaxial accelerometer and embedded gyroscope, the ActivPAL® and accompanying software quantifies time spent lying, sitting, upright and walking time, number of steps per day, cadence, and the amount of sit-to-stand and stand-to-sit transitions [22]. The activity monitors were applied at time of consent in the emergency department and removed prior to discharge. Corresponding with the day of patient inclusion, the monitor was set to record for 10 days nonstop, with the device in a non-latex waterproof sleeve and attached by a Tegaderm® dressing, placed midline on the quadriceps region of the nonfractured limb. To corroborate the activity monitor findings, we also employed a behaviour mapping technique to collect contextual data (e.g. environment, footwear, medical treatment), potentially influencing mobility activities. A research assistant observed the daily actions undertaken by study participants for each day the participant was in the study, between the hours of 8 am and 5 pm. Therapist and nursing notes were also reviewed for each day the participant was in the study.

In addition to the activity monitor and behaviour mapping data, we interviewed patients and their families to learn about their experiences with mobility activities during the first five days after surgery. The patient interview questions were grounded in the Capability, Opportunity, Motivation, Behaviour model (COM-B) of behaviour change [23]. This model proposes that behaviour change depends on an interacting system of these three components – capability, opportunity, and motivation. Using this model can help understand factors that influence mobility behaviours and potentially help identify a patient-focused intervention to improve these behaviours. The COM-B was selected as the behaviour change domains align with the TDF framework, and the two are often used in conjunction as a part of the Behaviour Change Wheel [19]. The constructs covered within the interview are supported in the literature relating to hospitalized older adults [24, 25].

Like the healthcare provider interview guide, the interviews were semi-structured, letting the natural flow of conversation allow for sharing of the patient’s experience. We strove to ensure the family interview question also inquired about patient-based activities. For example, within the Capability domain, a patient question was: thinking about how you felt *physically* right after your surgery – what do you think that you were capable (able) to do first 1–2 days (with or without help)?, whereas the family question wording would be – “thinking about how your (family member) appeared *physically* right after your surgery – what do you think that they were capable (able) to do first 1-2 days (with or without help)?” Interviews were conducted by one researcher (L.H), and took place at a time convenient to the patient and/or family member, prior to discharge. Each interview was audio recorded.

We obtained approval from both the hospital (REB 230–2018) and university (REH 730–18) health research Ethics Boards.

## Analysis

The overall analysis for a case study approach utilizes pattern matching, comparing known empirical evidence and predicted patterns to the study’s findings [13]. The objectives and design of the study have been based on the following propositions (predicted patterns):

1. Immediately following hip fracture surgical repair in the older adult (> 65), early mobility activities are limited [8, 26, 27]
2. Pre-fracture functional ability can be used to predict a patient’s outcome after hip fracture [28]
3. Patient, family, and healthcare provider preconceived notions influence mobility [29]

### Quantitative

Patient characteristics are described using descriptive statistics, including means and standard deviations. Behaviour mapping and narrative observational data is described using medians with a minimum to maximum range. R statistical software was utilized for activity monitor analysis, inclusive of data for any full days (i.e. 24 h) of movement activities commencing the first day after surgery (omitting pre-operative and intra-operative movement). For each patient, sedentary and upright times are described using means with a minimum to maximum range and standard deviations. Sit to stand transitions and steps taken are described using medians with a minimum to maximum range, as the data were not normally distributed. The maximum achieved value is highlighted across all data points. Group patterns and variations between pre-fracture functional status and cognitive status are reported.

### Qualitative

Patient and healthcare provider interviews were transcribed verbatim using Nvivo® transcription software, then checked for accuracy by the primary author. The interview narratives were analyzed line-by-line; firstly, the healthcare provider narratives, followed by the patient and family member narratives. The patient and family narratives were not separated by cognitive status nor by participant (i.e., we did not further divide the participants into sub-groups for analysis). We followed the thematic analysis approach to identify patterns across the dataset. Transcripts were read through several times (L.H.) to obtain a sense of the whole. L.H., V.D., and C.D. met to conduct line-by-line review of three healthcare provider transcripts to discuss the coding strategy and generate initial codes related to the study objectives. Each significant segment of the text received a conceptual code to classify and organize the information utilizing Nvivo® software. Coding was conducted via an inductive approach to identify themes and sub-themes. A codebook with definitions of themes and subthemes was generated, including theme name, the number of references, and sample quotes. The codebooks for healthcare providers and patient participants were then reviewed and compared for similarity amongst themes and identification of any new themes or subthemes.

In a convergent mixed methods design, after quantitative and qualitative data are collected, the data is first analyzed separately, followed by a side by side comparison of results to assess if the findings confirm or disconfirm each other [12]. Finally, the data merge and the results are presented together as a joint display [12].

## Results

### Recruitment

#### Main Case - Healthcare providers

The first author conducted individual face-to-face semi-structured interviews with consenting health care providers from May through to June 2019. There were ten individual health care provider interviews: two physiotherapists, two occupational therapists, one physiotherapy/occupational therapy assistant, and five registered nurses.

#### Embedded cases – patients

**Fig. 2:**
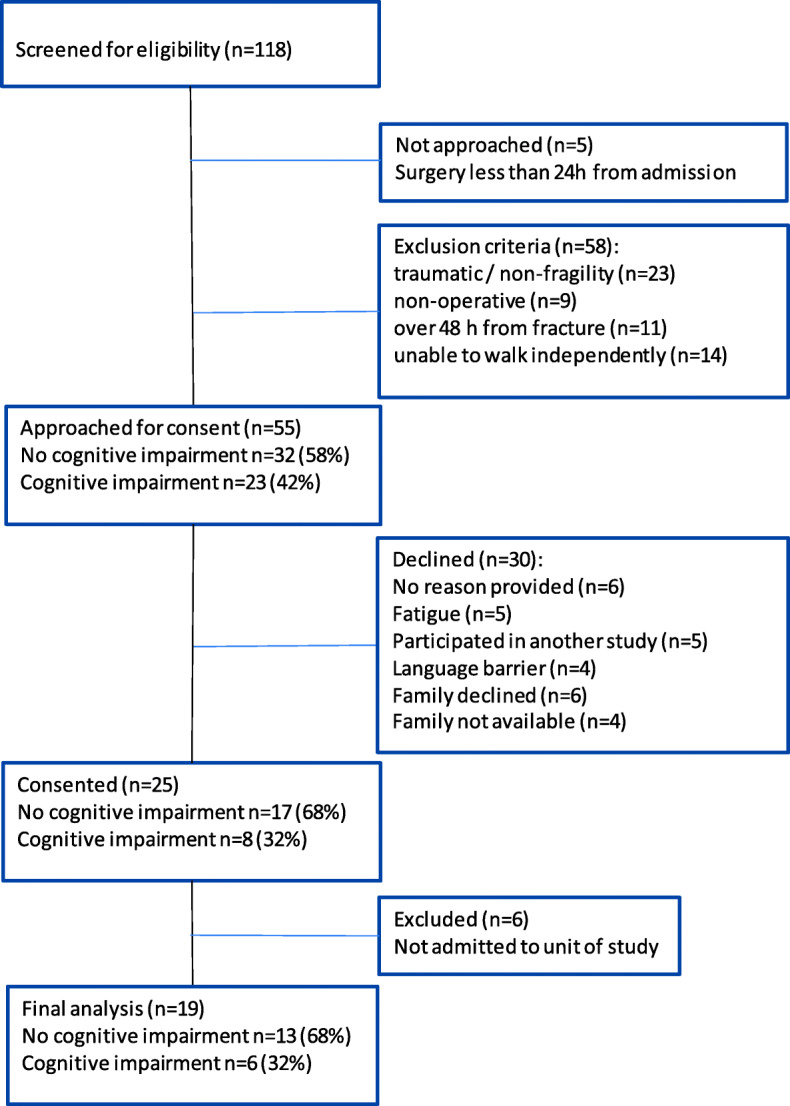
Flow diagram

Utilizing a convenience sampling approach, we consecutively screened 118 adults over 65 years of age admitted to the hospital secondary to a fragility hip fracture from November 2018 through to June 2019 (refer to Fig. 2: Flow diagram). Sixty-three patients were found to be eligible and approached for consent. Thirty-eight patients declined - reasons for not participating included fatigue, participation in another study, and presence of a language barrier. Interpretation services were not available when they were required.

Twenty-five patient-participants consented to participate in the study. As the research focuses on one post-operative unit, patients that were transferred to another unit after surgery were excluded (*n* = 6), leaving 19 patients for inclusion in the final analysis, of which six were identified to have cognitive impairment. The percentage of participants with cognitive impairment is representative of cognitive impairment prevalence in patients approached for consent (refer to Fig. 2: Flow diagram). There was a total of eighteen patient participant interviews: thirteen with patients only, three with family members, and two with both the patient and family member.

### Demographics

#### Main Case - Healthcare providers

Healthcare providers had all worked on the unit for over five years, except for one therapist (one year). The nursing staff consisted of all registered nurses (no registered practical nurses). Six of the ten (60%) healthcare providers were female. We did not obtain in-depth demographic information about the healthcare providers.

#### Embedded cases – patients

Patient participants ranged in age from 66 to 100 years, with an average age of 83.2 years (SD 10.5) (refer to Table 1: Patient Demographics). Fourteen (74%) participants were female. Fourteen (74%) participants lived in the community in an apartment/condominium or multi-story home. There were six patients (32%) with a cognitive impairment (two mild, four severe). In our cohort, cognitive impairment was associated with a low pre-fracture functional mobility; five of the seven (72%) patients with a low pre-fracture functional mobility also had a cognitive impairment. In the high pre-fracture functional group, there was one patient with mild cognitive impairment (MMSE 21).

**Table 1:**
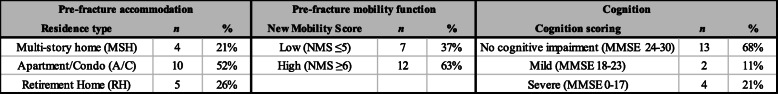
Patient Demographics (*n* = 19)

The mean length of stay (LOS) for the cohort was 11.4 days (SD = 12.3). There was a significant difference (*p* = .0007) between the mean LOS in those with a high pre-fracture functional mobility of 4.9 days (SD = 2.4), compared to the low group with a LOS of 22.5 (SD = 14.7).

### Activities and perceptions

#### Mobility related activity

We obtained complete activity monitor data from eighteen participants (refer to Table 2: Activity monitor data). The mean length of activity monitor wear time was 3.78 days (SD 1.08). The activity monitor data demonstrates a high mean daily sedentary time of 23.18 h, ranging from 17.9 to 24 h (SD 1.54). The median maximum upright time (standing, walking) was 24 min (Range 0.5–625), and the median number of maximum steps taken was 30 (Range 0–3762). Physiotherapy documentation was also reviewed to obtain a reported distance walked; the median maximum reported distance over the duration of stay was 12.5 m (Range 0–150 m).

**Table 2:**
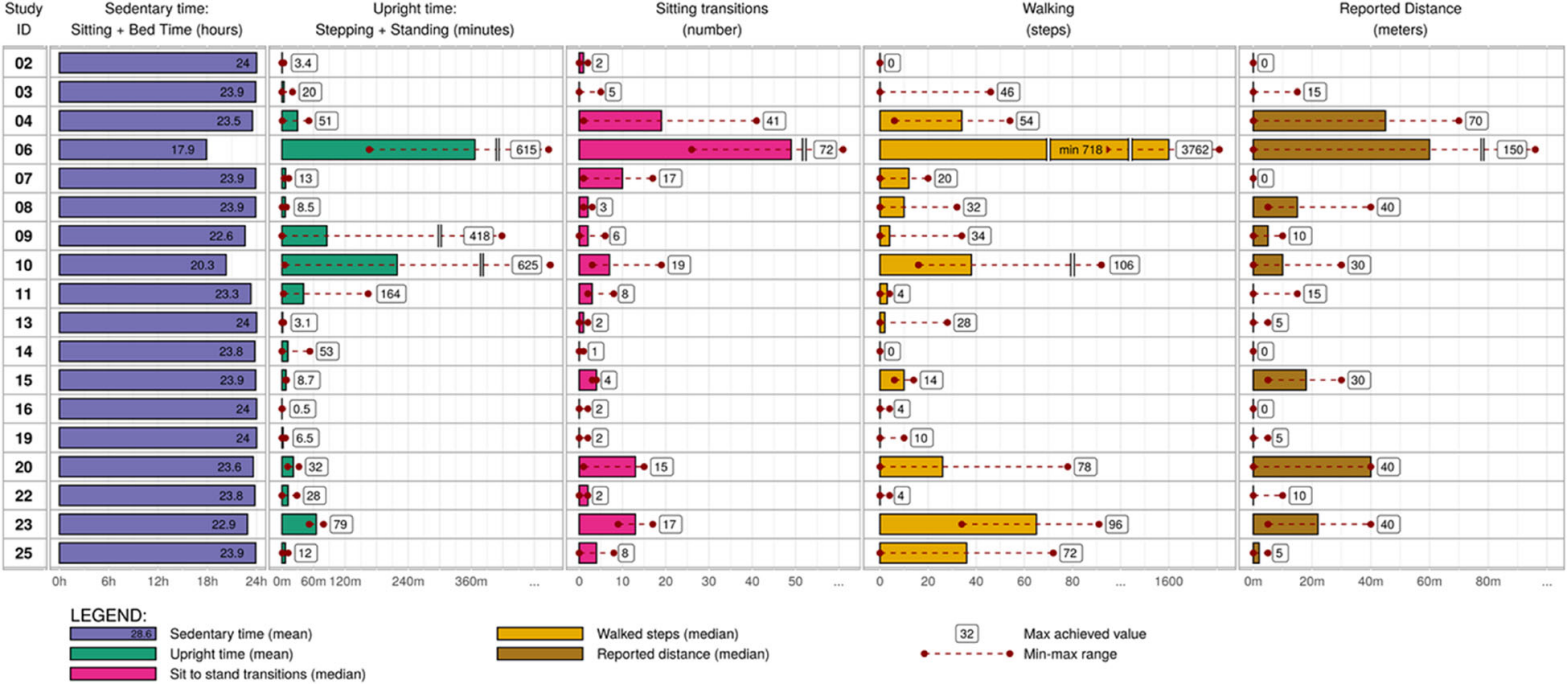
Activity monitor data (*n* = 18)

#### Perceptions

The qualitative interview analysis identified two main categories across all participants. The first category includes factors which are external to the patient, represented by two base themes; environment factors and healthcare provider perceptions. The healthcare provider theme had several sub-themes; ideal practice goals, attitudes and beliefs, prioritization of tasks, and perceptions of patient reactions to early mobility. The second category was identified as factors unique to each individual, represented by two base themes; psychological and physiological factors. The psychological theme was broken down into four sub-themes: fears (falling, re-injury, pain), sense of loss, trust in healthcare providers, and motivations. The physiological theme had two sub-themes: medical unpredictability and the need for rest.

### Factors associated with influencing participation in early mobility activities after surgery

**Fig. 3:**
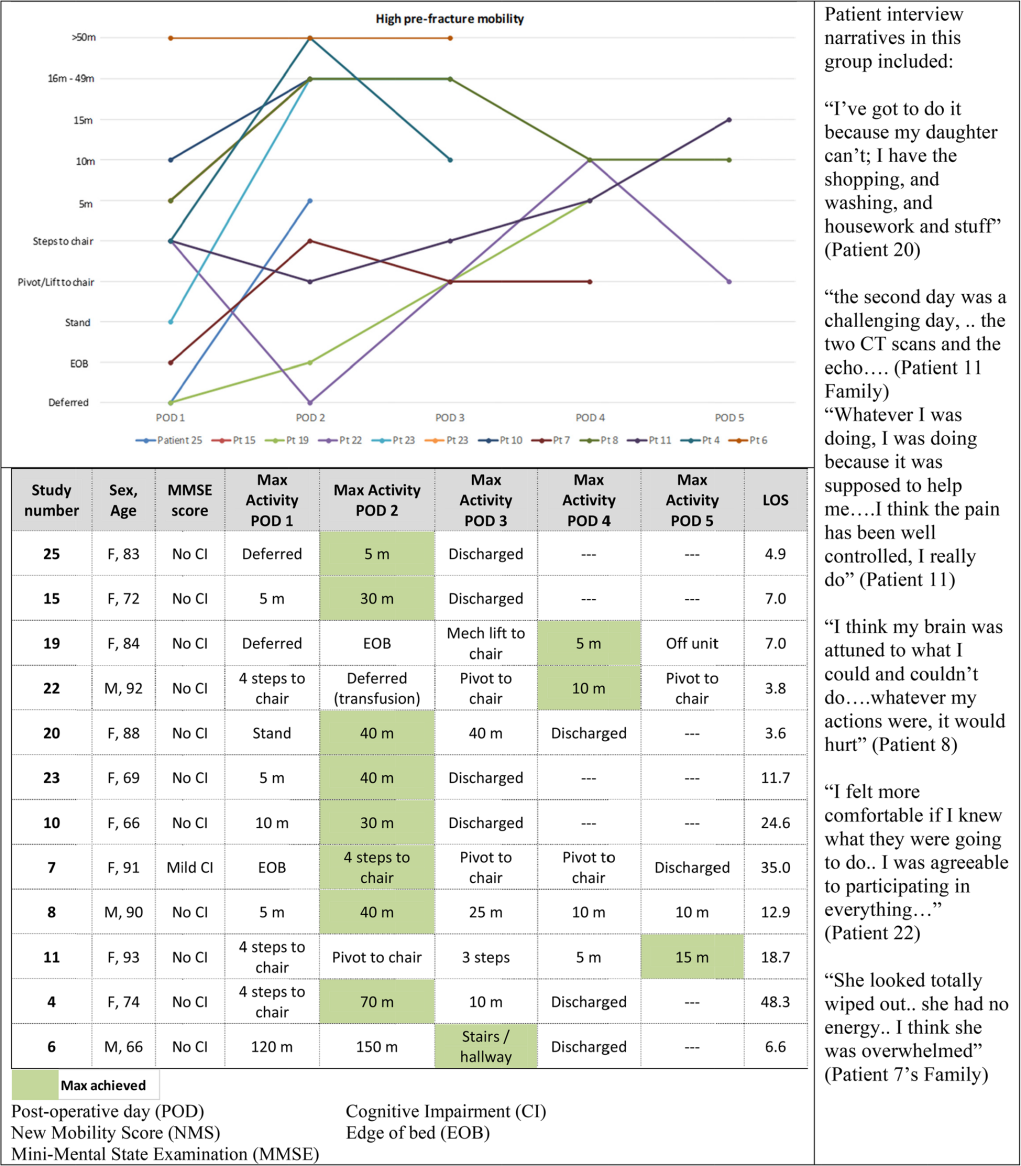
High pre-fracture mobility group. The line graph depicts the activities in the table

**Fig. 4:**
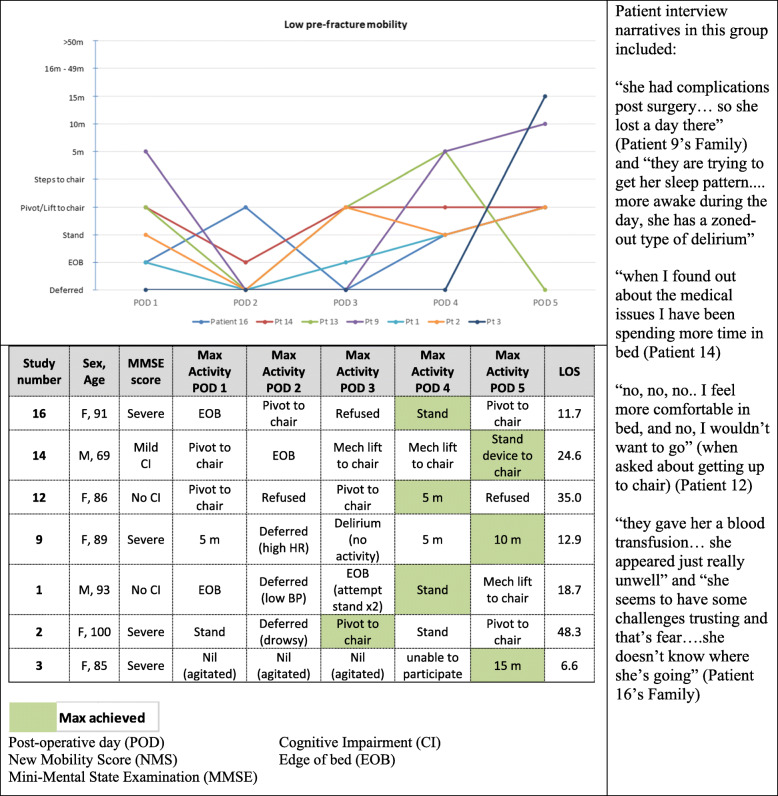
Low pre-fracture mobility group. The line graph depicts the activities in the table

We subsequently analyzed the quantitative and observational data alongside the qualitative themes. Sedentary times were notably high across all participants. The activity monitor data was stratified according to the pre-fracture functional ability as empirical evidence previously identified this is a factor that predicts outcomes (Figs. 3 and 4). In addition to pre-fracture function, we identified four other areas which have the potential to influence mobility; cognitive status, medical unpredictability (deferral of activities), healthcare provider attitudes and behaviours, and patient perceptions.

#### Pre-fracture function and post-surgical activity levels

Although there was a tendency for patients with high pre-fracture function (high NMS) (*n* = 12) to spend somewhat less time in sedentary behaviours (M = 22.9 h, SD =1.87) than patients with low pre-fracture function (low NMS) (*n* = 6) (M = 23.7 h, SD 0.50), these values were not significantly different (*p* = .16). However, there was a significant difference in the maximum reported daily walking distances between these two groups; 36.5 m for the high NMS group, compared to 5 m for the low NMS group (*p* = .04) (Figs. 3 and 4).

Five (42%) patients in the high NMS group walked to some degree on post-op day one, with one patient (Patient 6) walking 120 m. Eight of the twelve patients (67%) within this group achieved their maximum activity levels on post-operative day two.

In the low NMS group (Fig. 4) five of the seven patients (72%) also had cognitive impairment; and all four of the patient participants with a severe cognitive impairment were in this group. The maximum achieved activity was 10 m, and not achieved until post-operative day five. A healthcare provider deferring activity and participant non-participation was also high in this group.

#### Cognitive status

Patients with a low NMS and cognitive impairment (MMSE < 24) were less likely to achieve mobility within the first five days (Fig. 4). On the first day after surgery, most patients with cognitive impairment were at the edge of bed, standing, or walked within the room. During the following two days, these patients were more likely to be deferred secondary to medical issues or onset of delirium, many did not achieve their maximum levels of activity until day four or five. By the fifth day post-operatively, only two of the four patients with a severe cognitive impairment (Patients 3, 9) had walked. In the interviews, staff articulated that a multi-disciplinary approach was often required to mobilize this population “I think if someone has cognitive impairment I just wouldn’t mobilize them on my own” (RN1). Cognitive impairment was not always perceived to be a barrier by healthcare providers. Patient 9, a 90-year-old female with a severe level of cognitive impairment, was able to ambulate 5 m on the first day after surgery. “Sometimes they just have this automatic – they reach for the handles, and then, it’s more of an automatic thing” (PTA).

#### Medical unpredictability

A patient’s medical status was found to influence a healthcare providers’ decision to facilitate mobility. Healthcare providers stated “we have to focus more on acute illness first” (RN2) and often deferred mobility activities if they felt a patient was too medically well or unsafe for transfer: “Safety wise. .. . just looking for a red flag” (PT1). In our study, six (32%) patients’ activities were deferred over the first two days - three patients (16%) deferred on the first day, and an additional five patients (27%) on the second day due to varying acute medical conditions (Figs. 3 and 4). On the first day, two patients were deferred in the high NMS group, secondary to an electrolyte imbalance and severe pain. There was one deferral in the low NMS group secondary to severe agitation. On the second day, on deferral occurred in the high NMS group for a blood transfusion. In the low NMS group, deferrals were secondary to an increased heart rate, drowsiness, low blood pressure, and agitation.

Patients were reluctant to participate if the fall was precipitated by an underlying medical condition – Patient 19 shared ““I felt so weak, I had this vertigo for two or three days before I came in, …so I was still suffering from that”, requesting activities be deferred until her symptoms improved.

#### Healthcare providers attitudes and behaviours

All healthcare providers were aware of the hospital-based policies to promote early mobilization in fracture population - “we look beyond the hip fracture pathway, we also have the other senior friendly standards” (OT1). If staff were unable to implement their full plan for mobility, they articulated that they would compromise with an alternative to promote some activity and upright time; “….if they’re too ill to actually engage… they can still be hoyered *(sic)* up to chair” (PT2). This attitude was reflected in the HCP care behaviours for Patients 1, 14, and 19, who were mechanically assisted to chair as they were unable to stand or pivot themselves (Figs. 3 and 4). Similarly, healthcare providers articulated that they do expect a greater level of tolerance in those who are younger and/or have a higher pre-fracture functional ability. “If they’re mobile pre-op then the chance of getting them mobilized post-op is definitely higher. ... I would expect better results in those who are more active pre-op” (RN3).

#### Perceptions of early mobility

Many patients articulated that a hip fracture was a “serious injury” (Patient 22) and it would “take weeks to start feeling semi-normal” (Patient 25) and “will take longer than that to really get back” (Patient 23). They did not expect to be mobile in the first few days – “it would be a few days before I could do anything” (Patient 20). Patients reported that they felt the timing and duration of mobility activities was up to the healthcare providers, even if they felt they would have been able to tolerate more. As an example, Patient 4 stated “I thought I probably could have done more, but I didn’t know how much I should do, I left it to them”, she had a documented distance of 70 m on POD 2 with physiotherapy, but only 10 m the following day as she ambulated with nursing (Table 2).

Healthcare providers and patient participants both reported that a fear of falling, fear of pain, or fear of reinjury were potential factors having an impact on the ability to participate: “... .pain control is the number one issue but then there’s anxiety on top of that – sometimes they are just afraid to move” (PT2). Patient 25 shared “I was really sweating bullets…. I was in pain and nothing was moving and I was scared of damaging it”. She was only able to ambulate 5 m on her second day, with her activity monitor reporting 72 steps taken (Table 2).

Some patients reported how experiencing a fall and fracture has impacted their future; specifically some patients talked about experiencing a sense of loss – a potential loss of ability, or change in pre-fracture functional mobility; for example Patient 7 stated “I couldn’t do anything, you would sit and think of all the things you couldn’t do anymore; I will never not be ‘overwhelmed’ at what has happened. I can see myself laying on that floor… I won’t forget that easily.” This sense of being overwhelmed may have impacted her ability to mobilize; her activity monitor demonstrated a maximum of 20 steps (Table 2). Conversely, often discussed the need to resume ‘normal’ life and find reasons to motivate or push themselves for quick recovery; sometimes these motivators were identified as ongoing responsibilities: Patient 8 stated “my main thinking is how can I manage my own problems and help my wife at the same time, that is my main concern.” Patient 25 was an early ambulator, she articulated that “I have to get back to being active very fast. I’ve got an unwell husband at home.”

## Discussion

This study utilized an embedded case study design to represent a post-operative unit as the main case and older adults recovering from hip fracture as embedded cases. Collection of quantitative data with activity monitors and concurrent qualitative interviews with patients, family members, and healthcare providers allowed for a comprehensive portrayal of the phenomenon of interest. The subsequent convergent analysis of all data identified locally relevant factors that influence mobility activities. To our knowledge, this is the first study that examines both the patients and their environment (inclusive of healthcare providers, physical environment and health care processes) to identify factors influencing participation in early mobility post-fracture.

Returning to the propositions, the first proposition is an acknowledgement of previous findings in the literature that early mobility activities are limited immediately following hip fracture surgical repair in older adults (> 65) [8, 26, 27]. In alignment with this evidence, our patient sample was observed to have a very high sedentary time (lying or sitting), with a mean of 23.8 h in bed. Except for one patient, a very small number of steps were taken during the acute care stay. The potential explanation for this limited activity within the hospital setting proved to be multi-layered and complex, influenced by several factors: specifically, pre-fracture functional status, cognitive status, perceived health status, healthcare provider attitudes and beliefs, and patient perceptions.

The second proposition highlighted that a patient’s pre-fracture functional ability can be used to predict patient outcomes after hip fracture [28]. When the patient data was stratified by pre-fracture functional ability, mobility patterns became evident. In our cohort, those with a high pre-fracture functional ability were able to engage more consistently in higher intensity activities. Healthcare providers also identified a belief that pre-fracture functional ability is an influencing factor in the interviews - staff members articulated that if they were independently mobile pre-operatively that “chances of them getting mobilized post-op is definitely higher.” Most of the patients with a higher pre-fracture functional ability achieved their maximum activities sooner – often by the second post-operative day, compared to the fourth post-operative day in patients with a low pre-fracture functional ability. Similarly, the activities were more intense, for example, walking out of the room occurred frequently in this group. The high pre-fracture functional group was also discharged to rehabilitation significantly faster. Pre-fracture functional ability has been previously identified as a predictor of ability to regain mobility while in hospital by several studies [28]. Education and encouragement for patients with a high pre-fracture functional ability may be all that is required in this population, as evidence demonstrates this group is more likely to reach their baseline functional goals.

Conversely, the literature has identified those with a low pre-fracture functional ability are at highest risk of not regaining basic mobility and are at risk of institutionalization [28]. The reasons for this are likely complex, but one influencing factor we identified was cognition. Most of our cohort with a low pre-fracture functional ability also had cognitive impairment. There are a number of gait changes in those who have cognitive impairment (CI), which places them at a substantially higher risk of sustaining a hip fracture than those who are cognitively intact [30–32]. After a physiological stressor, such as a fall or a surgical procedure, older adults with underlying cognitive frailty are more likely to develop cognitive complications such as delirium [30, 33]. Studies have demonstrated that patients with lower cognitive function are at higher risk for poorer functional outcomes [34, 35], are less frequently transferred to rehabilitation clinics [34], and are more likely to become institutionalized [36, 37]. Additionally, the presence of a cognitive impairment has been identified as a potential factor that deters patients from participating in early mobility activities. Previous research has identified that memory problems, poor insight, and loss of purposeful movements have been a barrier to engaging this sub-population in activities [38]. The low pre-fracture functional ability, especially patients with cognitive impairment, would benefit from specialized care from a provider aware of issues in this sub-population [15]. To date, there is no evidence to direct the content, duration and frequency of physiotherapy treatment, or the optimal approach to mobility for the interdisciplinary team in patients with low pre-fracture functional status, indicating a need for future research [39].

The last proposition recognizes that healthcare provider and patient and family preconceived notions influence mobility [29]. One significant notion held by healthcare providers was the belief that they need to “focus more on acute illness first”, subsequently deferring mobility activities until medical stability could be achieved. In the first two post-operative days, six (32%) of our study patients had their mobility deferred; making this a potential area for behaviour change interventions. Safety concerns about patients engaging in early mobility are not uncommon, in a critical care study, nurses identified that patient safety concerns such as hemodynamic instability, orthostatic hypotension and fear of falling or injury represented the most significant barrier to mobilization [40]. LA Kimmel, SM Liew, et al. [41] demonstrated that an intensive physiotherapy program for hip fractures is safe and can reduce length of stay. For those who are more acutely ill, data from critical care studies that identify mobilization activities can be safe and feasible in more acutely ill critical care patients, with a relatively small number of adverse events reported (< 1%) [42].

A healthcare providers’ notion about expectations for recovery can also influence a patients’ mobility activities. Healthcare providers shared that the system is ‘discharge backward’. Healthcare providers prioritize their assessments to expedite completion of discharge rehabilitation applications, enabling a timely discharge to a rehabilitation setting for those who can participate in early mobility activities. This was demonstrated in our cohort - patients who ambulated the first day (high NMS) were more likely to be discharged within five days. Patients who have longer lengths of stay, and no longer on the expedited care pathway, are lower on a therapist provider’s priority.

There is the belief from therapists that ongoing mobility will continue once initial assessments and treatment plans have been formulated. For all patients, therapists shared that once a mobility plan has been set out, it is an expectation that the nursing team and therapist assistant then carry out the mobility plan, which may not include intensity or frequency in progression of activities. However, nurses articulated their primary goal is to get a patient up to a chair once daily – a low intensity activity. The lack of mobility activity progression may subtly communicate to patients that sitting in a chair is an adequate activity for recovery.

An increased risk of poor outcomes occurs by compounding multiple factors, such as a patient with low pre-fracture functional mobility, cognitive impairment, and a healthcare provider’s beliefs and systemic expectations. This population is more likely to experience subsequent prolonged periods of bedrest, placing them at increased risk of institutionalization secondary to functional decline [3]. Ultimately, this is the patient population that should be the target of any future research and intervention implementation.

Similar to healthcare provider notions – a patient’s preconceived notion influences their participation in mobility activities. Our study has identified the ‘mismatch’ of recovery expectations; patients explained the need for ample rest to avoid physical exhaustion, whereas healthcare providers considered active mobilization as the key strategy to expedite recovery. CJ Brown, BR Williams, et al. (2007) also identified that patients often assume bedrest during hospitalization is a necessity. Several patients within our cohort expressed that they “need time to recover” and were not expecting to ambulate so quickly after surgery. Many patients believed that it would take a longer time to recover as they were older, inferring that rest would be required, given their age. An alternative explanation of those who were able to do more is the concept of “I left it to them” in which patients expressed that they felt they could have done more, but they were trusting that the health care providers knew what their personal limitations were, and what they were supposed to achieve to transition to a rehabilitation facility. How each healthcare provider bridges this gap between expectations is the challenge and opportunity for future interventions.

Lastly, patients expressed that it is not only physical capability, but psychological readiness that may be a barrier. Several patients articulated that they were afraid they would ‘break something’ or that they were afraid of incurring pain. Previous studies have reported similar findings [43, 44]. A priority for engagement in mobility after surgery is a pain management approach that is communicated clearly to patients and healthcare providers.

## Limitations

We recognize that a limitation is within the scope of the study; we intended the study to closely examine practices of one unit, the findings may not be generalizable to all. However, our activity monitor data is in keeping with previous published studies [9, 26]. A limitation of the ActivPAL® monitor is the limited ability to accurately detect steps taken at a slower walking speed (< 0.45 m.sˉ^1^) [22]. The walking speed limitation may be relevant to this older adult population recovering after surgery, and so these data were supplemented with observational data collected via behaviour mapping and a review of therapists’ narrative regarding patient performance (e.g. distance walked) during therapy within the chart.

In those with a cognitive impairment, timely contact with substitute decision makers proved to be a challenge; there were seven eligible patients that we could not approach for informed consent as we were unable to reach their family members. However, on review of characteristics of patients included and those excluded or missed, we demonstrate that our numbers of participation in those with cognitive impairment was likely representative of the presenting population. In an effort to be inclusive of patients with cognitive impairment, we interviewed family members of those patients unable to participate in the interview themselves. The perceptions related to early mobility of these individuals acting as proxies may not accurately reflect the patient experience.

## Conclusion

In conclusion, we found that there are high sedentary times after surgical repair for fragility hip fracture. We have identified in-hospital contextual factors which are important considerations for facilitation of early mobility by healthcare providers and participation by patients. To address the mismatch of expectations between patients and providers, hospitals may consider additional opportunities for integration of patient education about expectations for mobility after surgery. There is a need to recognize and act upon the risk of poor outcomes in the sub-population of hip fractures that present with multiple risk factors (low pre-fracture function and cognitive impairment, and medical unpredictability). Integration of documentation of a patient’s pre-fracture functional status and identification of cognitive impairment on admission can potentially lead to enhanced post-operative care that encourages greater mobility in this population. As stated, there are no studies that examine differing treatments to maintain baseline functional status in those with a lower pre-functional ability or cognitive impairment. Given the lack of data available for perioperative care in the subpopulation of patients with hip fracture and cognitive impairment, this is an essential area for future research to improve their outcomes.

## Data Availability

The datasets during and/or analyzed during the current study available from the corresponding author on reasonable request.

## Abbreviations

CI: Cognitive Impairment
COM-B: Capability, Opportunity, Motivation for Behaviour Change
HCP: Healthcare provider(s)
LOS: Length of stay
MMSE: Mini Mental State Examination
NMS: New Mobility Score
OT: Occupational therapist
POD: Post-operative day
PTA: Physiotherapist assistant
PT: Physiotherapist
RN: Registered nurse
TDF: Theoretical domains framework
